# Pyroptotic Macrophage-Derived Microvesicles Accelerate Formation of Neutrophil Extracellular Traps *via* GSDMD-N-expressing Mitochondrial Transfer during Sepsis

**DOI:** 10.7150/ijbs.87646

**Published:** 2024-01-01

**Authors:** Liangjian Kuang, Yongjian Wu, Jingxian Shu, Jingwen Yang, Haibo Zhou, Xi Huang

**Affiliations:** 1 Center for Infection and Immunity and Guangdong Provincial Key Laboratory of Biomedical Imaging, The Fifth Affiliated Hospital of Sun Yat-sen University, Zhuhai, Guangdong Province, 519000, China.; 2 The Sixth Affiliated Hospital of Guangzhou Medical University, Qingyuan People's Hospital, Qingyuan, Guangdong Province, 511518, China.

**Keywords:** Pyroptosis, microvesicles, neutrophil extracellular traps, mitochondrial transfer, sepsis

## Abstract

Macrophage pyroptosis and neutrophil extracellular traps (NETs) play a critical role in sepsis pathophysiology; however, the role of macrophage pyroptosis in the regulation of NETs formation during sepsis is unknown. Here, we showed that macrophages transfer mitochondria to neutrophils through microvesicles following pyroptosis; this process induces mitochondrial dysfunction and triggers the induction of NETs formation through mitochondrial reactive oxygen species (mtROS)/Gasdermin D (GSDMD) axis. These pyroptotic macrophage-derived microvesicles can induce tissues damage, coagulation, and NETs formation *in vivo*. Disulfiram partly inhibits these effects in a mouse model of sepsis. Pyroptotic macrophage-derived microvesicles induce NETs formation through mitochondrial transfer, both *in vitro* and *in vivo*. Microvesicles-mediated NETs formation depends on the presence of GSDMD-N-expressing mitochondria in the microvesicles. This study elucidates a microvesicles-based pathway for NETs formation during sepsis and proposes a microvesicles-based intervention measure for sepsis management.

## Introduction

Sepsis is life-threatening; it is characterized by a dysregulated host inflammatory response to infection that causes severe inflammation, organ dysfunction, and various types of cellular damage**1-4**. Sepsis is the reason for the highest mortality in intensive care unit (ICU) patients. Treatment techniques for sepsis have advanced significantly over the past 20 years; however, there are still no effective drugs to combat sepsis. Therefore, it is critical to evaluate the pathogenesis of sepsis for identifying new targets for treatment.

Neutrophil extracellular traps (NETs) formation is a special form of neutrophil death **5**, in which DNA, myeloperoxidase (MPO), and neutrophil protease (NE) are released as a net-like structure**6, 7**. NETs facilitate the progression of diseases, such as COVID-19**8**, thrombosis **9**, and cancer **10**. NETs play a critical role in sepsis**11, 12** , and the presence of NETs can be used to predict mortality in sepsis patients**13**. However, the factors involved in the regulation of NETs formation are largely overlooked when assessing the role of NETs formation in sepsis.

Pyroptosis of macrophages plays an important role in antibacterial immunity and lethal endotoxemia**14-16**. Following the activation of inflammatory caspase-1 and caspase-11, the pore-forming protein, gasdermin D (GSDMD), is cleaved, in the macrophages. This cleavage allows their oligomerization to form pores in the plasma membrane. IL-1β, IL-18, and damage-associated molecular patterns are released; these contribute to the inflammatory response and cytokines storm **17.** However, whether macrophage pyroptosis contributes to NETs formation and the underlying mechanism of sepsis is largely unknown.

Microvesicles (MVs) are membrane-derived vesicles of submicron diameter; they are released by many cells upon stimulation or apoptosis. These MVs express membrane proteins derived from their cell of origin**18.** Sepsis is associated with microvesicles from different cell types. Pyroptotic macrophage-derived MVs can trigger blood clotting and host death during sepsis**19**. In addition, the exosomes from active platelets obtained via the Akt/mTOR autophagy pathway induce NETs formation in sepsis**20.** The high plasmin generation capacity (PGC) of granulocyte MVs (Gran-MVs) reduces the formation of thrombuses and increases survival during sepsis**21**. Mitochondria can transfer between different cells through extracellular vesicles (EVs); this transfer influences the biological function of recipient cells. Mesenchymal stromal cell (MSC)-derived mitochondria are transferred to pulmonary epithelial cells through EVs; this decreases inflammation and injury in a mouse model of acute lung injury **22**. In addition, activated platelets can deliver respiratory-competent mitochondria to MSCs, and improve their wound-healing capacity **23**. Activated monocytes release mitochondria through EVs; these induce an inflammatory response in target cells **24**. However, whether the dysfunctional mitochondria of pyroptotic macrophages can transfer via MVs and regulate sepsis progress remains to be elucidated.

In this study, we observed that neutrophils could endocytose the MVs released from pyroptotic macrophages. These mitochondria could transfer from pyroptotic macrophages to neutrophils through MVs; this induced NETs formation and ROS production. In addition, MVs could induce mitochondrial dysfunction in neutrophils and trigger the mtROS/GSDMD axis, which lead to NETs formation. The presence of GSDMD-N-expressing mitochondria in pyroptotic macrophage-derived microvesicles is a major factor regulating NETs formation and altering mitochondrial homeostasis in neutrophils. We administered pyroptotic macrophage-derived MVs into mice and observed organ damage and systemic coagulation *in vivo*. MVs from bronchoalveolar lavage fluids (BALFs) of sepsis or acute lung injury (ALI) models induced NETs formation, which was partly inhibited by the GSDMD inhibitor, disulfiram. Therefore, this study elucidates novel mechanisms of NETs formation during sepsis and could provide novel therapeutic strategies.

## Results

### MVs from pyroptotic macrophages contained more mitochondria

**Figure 1:**
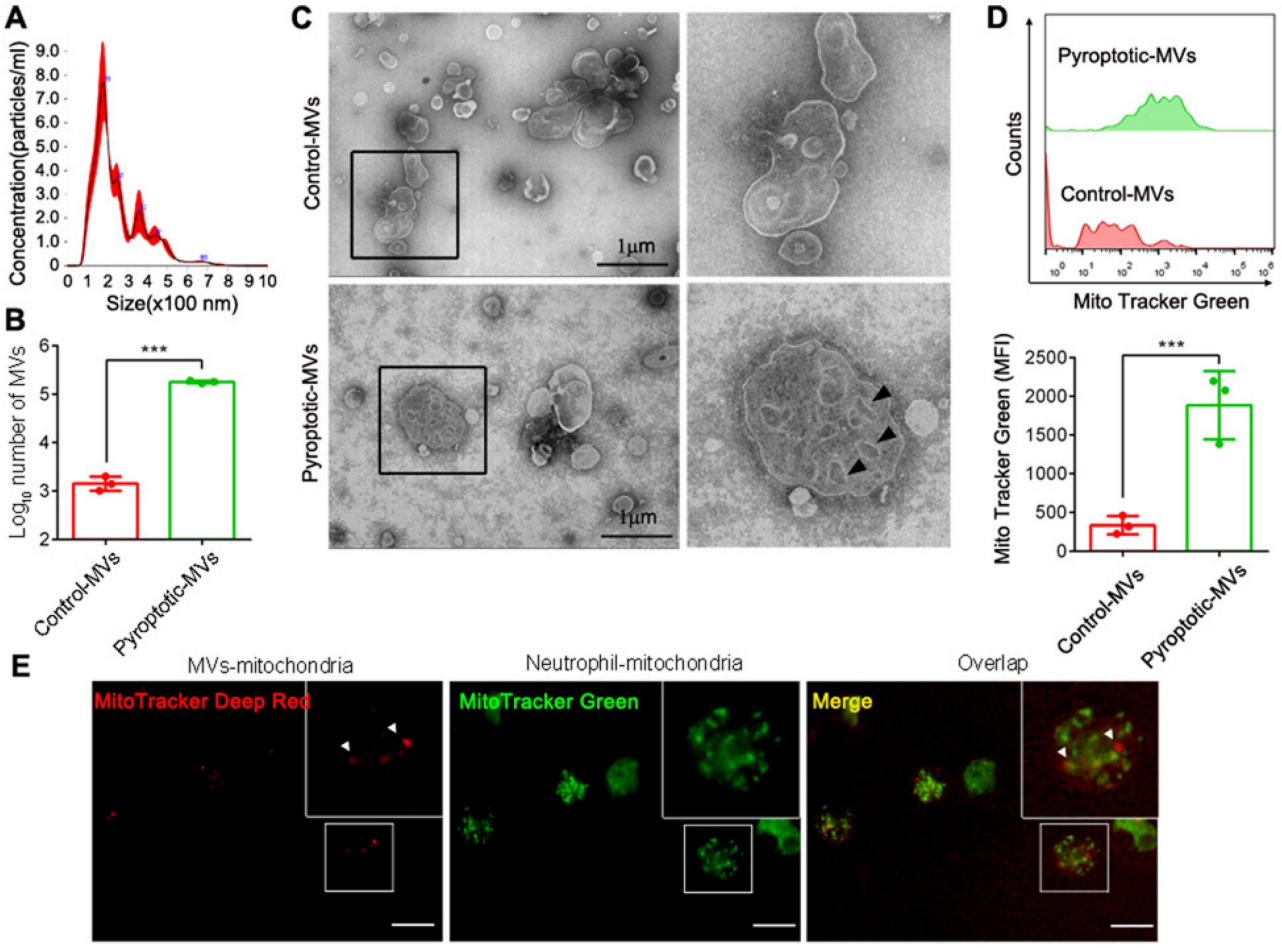
** Characterization of microvesicles after isolation and internalization in neutrophils.** (A) Representative image of NTA. (B) The production of MVs from macrophages that underwent pyroptosis or not. (n=3 wells per group). (C) Representative TEM images showing: multivesicular bodies from control-MVs (upper, scale bar, 1 µm) and mitochondria-like structures from pyroptotic-MVs (down, scale bar, 1 µm). (D) Representative flow cytometry images showing mitochondria in MVs between pyroptotic macrophage-derived MVs (pyroptotic-MVs) and control macrophage-derived MVs (control-MVs) (n=3 wells per group). (E) Representative fluorescent microscopy images of pyroptotic-MV mitochondria internalization in neutrophils (mitochondria from MVs (red), mitochondria from Neutrophil (green), scale bars, 20 µm). Statistical analysis performed using unpaired Student's *t*-test. Results are expressed as mean ±SEM.

MVs contain mitochondria**22, 23**. To assess whether pyroptotic macrophage-derived MVs contained mitochondria, we used nanoparticle tracking analyses (NTAs) to demonstrate that the particle size of these MVs was in the range of 100-1000 nm **(Fig. 1A)**. To demonstrate the difference in the MVs between control macrophages and pyroptotic macrophages, we subjected the same number of cells (4×10^6^ THP1) to pyroptosis or not, and used flow cytometry to examine MVs production and for the presence of mitochondria. Pyroptotic macrophages produced more MVs than the control macrophages **(Fig. 1B)**. These MVs had mitochondria-like morphology, as observed using transmission electron microscopy (TEM)** (Fig. 1C)**. In addition, the pyroptotic macrophage MVs contained more mitochondria than the control macrophages** (Fig. 1D and Supplemental 1)**. We investigated whether pyroptotic macrophages could transfer mitochondria to neutrophils through MVs. We stained for the different mitochondrial markers; pyroptotic macrophage-derived MVs were taken up by neutrophils **(Fig. 1 E),** suggesting that the mitochondria were transferred via MVs.

### Pyroptotic macrophage-derived MVs induced NETs formation and increased the production of ROS *in vitro*

**Figure 2:**
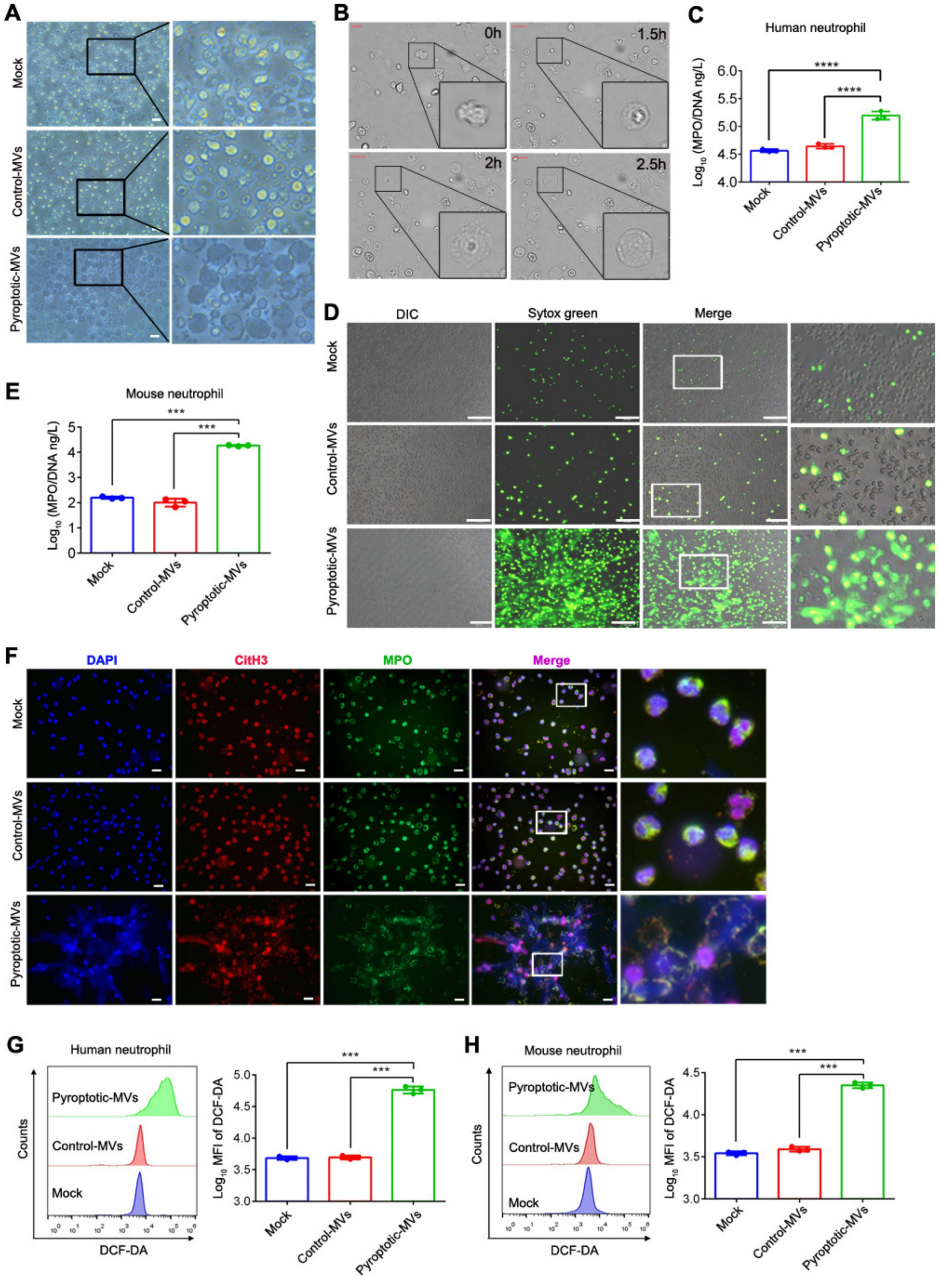
** Pyroptotic macrophage-derived MVs induce NETs formation and ROS Production.** Human peripheral neutrophils and mouse bone marrow neutrophils were isolated and cultured with PBS, control macrophage-derived MVs, or pyroptotic macrophage-derived MVs for 4 h at 37°C. (A) Morphology of human peripheral neutrophils. Scale bar, 20 μm. (B) Live-cell images of human peripheral neutrophils after treatment with pyroptotic macrophage-derived MVs. (C) The concentration of MPO-DNA complexes released by human peripheral neutrophils was assessed using ELISA. (n=3 wells per group). (D) Representative Sytox Green fluorescence image for NETs formation of human peripheral neutrophils. (n=3 wells per group) (E.) The concentration of MPO-DNA complexes released by mouse bone marrow neutrophils assessed using ELISA. (n=3 wells per group). (F) Representative immunostaining images for DNA (DAPI, blue), myeloperoxidase (MPO, green), and the citH3 (red) of human peripheral neutrophils. (G and H) Reactive oxygen species generated by human peripheral neutrophils (G) and bone marrow neutrophils (H) assessed using 2,7,-dichlorofluorescein diacetate (DCF-DA) staining. (n=3 wells per group). Results are represented as mean ±SEM.

NETs formation and ROS production can damage organs**8, 25**; therefore, we aimed to determine whether pyroptotic macrophage-derived MVs could induce NETs formation and increase ROS production *in vitro*. Human and mouse neutrophils were exposed to pyroptotic macrophage-derived MVs for 4 h; the neutrophil morphology changed, and they exhibited swelling followed by rupture. This was similar to the response of neutrophils treated with phorbol 12-myristate 13-acetate (PMA) **(Fig. 2A and Supplemental 2A)**. In addition, we used live cell imaging for dynamic monitoring of cellular changes; neutrophils swelled and ruptured following MVs treatment **(Fig. 2B and Supplemental movie 1)**. To determine whether neutrophils can undergo NETosis in response to treatment with pyroptotic macrophage-derived MVs, we used an* in vitro* co-culture assay of neutrophils plus PBS, control macrophage-derived MVs, or pyroptotic macrophage-derived MVs. We used an enzyme linked immuno-sorbent assay (ELISA) to assess NETs formation; the amount of MPO-DNA complexes was significantly higher in the supernatants from co-cultures of human and mouse neutrophils with pyroptotic macrophage-derived MVs compared to that with PBS or control macrophage-derived MVs **(Fig. 2C and E)**. The cell-impermeable DNA dye, Sytox Green, was used to assess DNA release from neutrophils**26**. After 3-4 h of co-culture with PBS or control macrophage-derived MVs, there was no extracellular DNA detected, suggesting that there were no NETs present. However, extracellular DNA was detected in co-cultures with pyroptotic macrophage-derived MVs **(Fig. 2D and Supplemental 2B).** To confirm that the extracellular DNA originated from NETs, we stained for the granule proteins, MPO and citrullinated histones (citH3), which are specific NETs markers**7**. The expression of MPO and citH3 was increased in human and mouse neutrophils co-cultured with pyroptotic macrophage-derived MVs compared to that with control macrophage-derived MVs or PBS **(Fig. 2F and Supplemental 2C)**. To exclude the effect of other immune cell on NETs formation, the purified neutrophils were sorted from peritoneal lavage fluids **(Fig. Supplemental 3A)**, and then exposed to pyroptotic macrophage-derived MVs or control macrophage-derived MVs for 4 h; and Sytox Green was used to detect the NET formation. Consistent with previous results, the purified neutrophils stimulated with pyroptotic macrophage-derived MVs has excess NET formation compared with macrophage-derived MVs **(Fig. Supplemental 3B)**. Therefore, pyroptotic macrophage-derived MVs can induce NETs formation; this was not the case with the control macrophage-derived MVs. Next, we explored the role of pyroptotic macrophage-derived MVs in ROS production in neutrophils. When neutrophils were exposed to pyroptotic macrophage-derived MVs, control macrophage-derived MVs or PBS, ROS production was significantly higher in neutrophils exposed to pyroptotic macrophage-derived MVs compared to that with control macrophage-derived MVs or PBS **(Fig. 2J and K)**. Therefore, pyroptotic macrophage-derived MVs have the capacity to increase NETs formation and ROS production.

### Pyroptotic macrophage-derived MVs altered mitochondrial homeostasis in neutrophils

**Figure 3:**
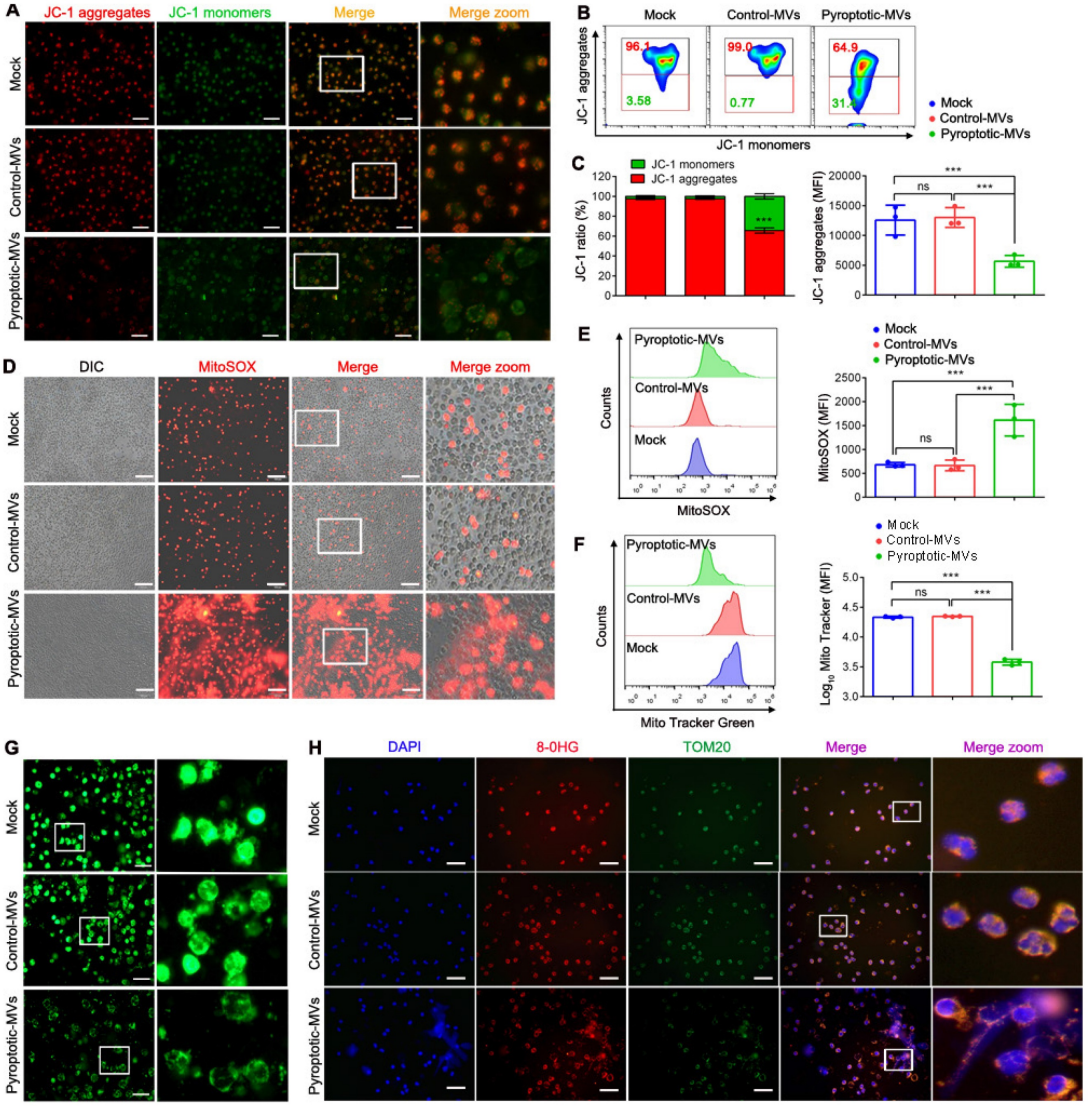
** Pyroptotic Macrophage-derived MVs Altered Mitochondrial Homeostasis in Neutrophils.** Human peripheral neutrophils were isolated and cultured with PBS, control macrophage-derived MVs, or pyroptotic macrophage-derived MVs. (A, B and C) ΔΨ of human peripheral neutrophils was assessed using JC-1staining. JC-1 monomers (green) and aggregates (red) were assessed using fluorescence microscopy (A) and flow cytometry (B and C). Scale bar, 20 µm. (D and E) MitoSOX of human peripheral neutrophils was assessed using fluorescence microscopy (D) and flow cytometry (E). Scale bar, 50 µm. (F and G) Mitochondrial mass in human peripheral neutrophils was detected using flow cytometry (F) and fluorescence microscopy (G). Scale bar, 20 µm. (H) Representative images showing neutrophil staining of DNA (DAPI, blue), TOM20 (green), and 8-OHG (red) in human peripheral neutrophils following exposure to MVs for 4 h. Results are represented as mean ± SEM.

The mitochondria play an important role in NETs formation **27**; therefore, we assessed whether the MVs altered the neutrophil mitochondrial homeostasis. The mitochondrial membrane potential of human neutrophils was markedly decreased following treatment with pyroptotic macrophage-derived MVs, compared to that with the PBS or control macrophage-derived MVs, as determined by immunofluorescence and flow cytometry **(Fig. 3A- C)**. In addition, the level of mitochondrial reactive oxygen species (mtROS) was significantly increased **(Fig. 3D- E)**; there was a significant reduction in the number of mitochondria **(Fig. 3F - G)**. Similar results were observed in mouse neutrophils **(Fig. Supplemental 4 A-B)**. We evaluated MVs-induced DNA oxidation through staining with 8-oxo-2'-deoxyguanosine (8-OHdG)-specific antibodies, for understanding the potential adverse effects of mtROS. Following treatment with pyroptotic macrophage-derived MVs, the neutrophil cell surface and the extruded NETs were strongly stained with 8-OHdG** (Fig. 3H)**. TOM20 antibodies co-localized with 8-OHdG during immunofluorescence, indicating that most of the oxidation occurred in the mitochondria and not in the chromosomal DNA. Therefore, pyroptotic macrophage-derived MVs altered mitochondrial homeostasis in neutrophils.

### Pyroptotic macrophage-derived MVs trigger mtROS/ GSDMD axis in human neutrophils

**Figure 4:**
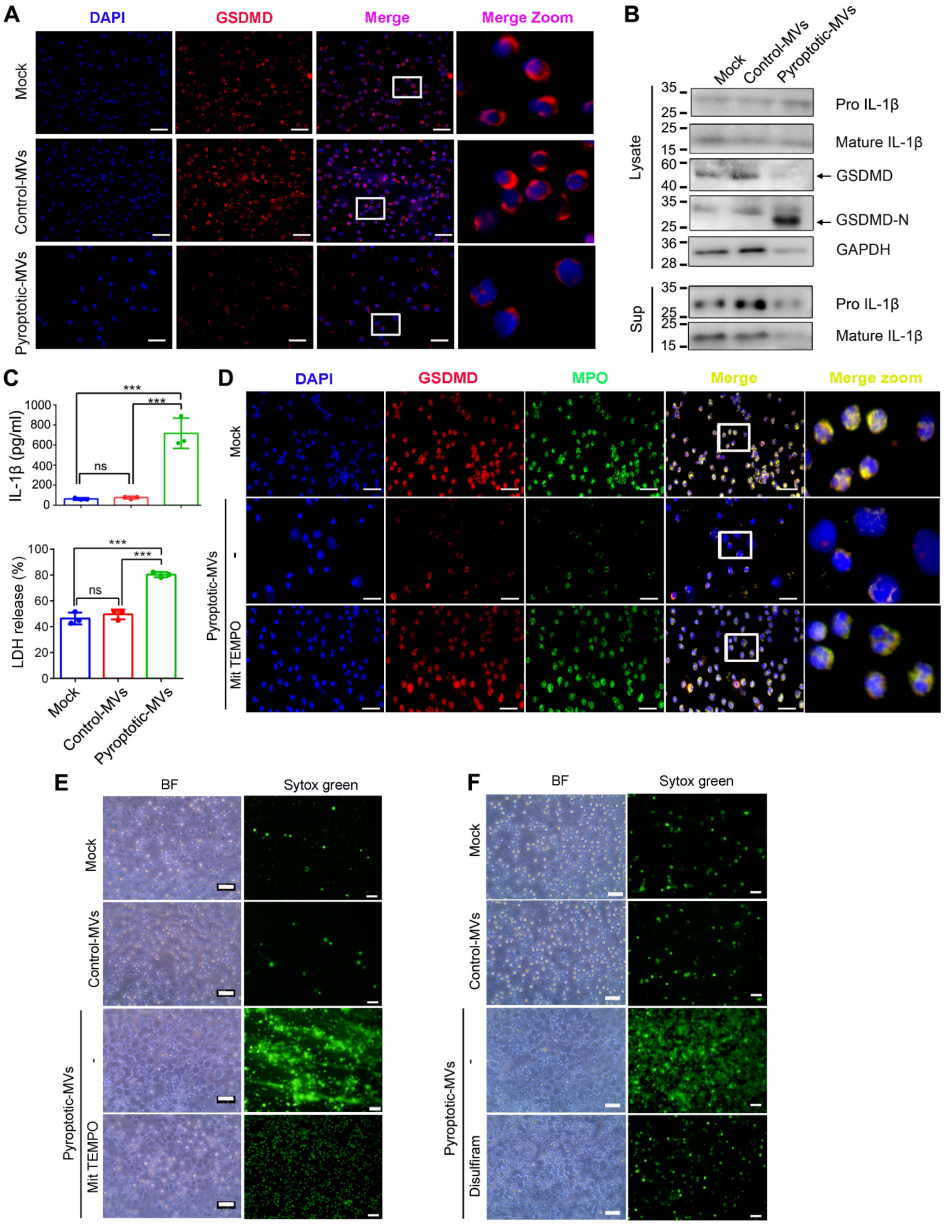
** Pyroptotic Macrophage-derived MVs Trigger mtROS/ GSDMD axis to Induce NETs Formation.** Human peripheral neutrophils were isolated and cultured with PBS, control macrophage-derived MVs, or pyroptotic macrophage-derived MVs. (A) Representative images showing neutrophil staining of DNA (DAPI, blue) and GSDMD (red) in human peripheral neutrophils after exposure to MVs for 4 h. Scale bar, 20 µm. (n=3 wells per group). (B) Human peripheral neutrophils were stimulated with MVs for 2 h, and the expression of GSDMD, GSDMD-N, Pro- and cleaved IL-1β in cell lysates and supernatants was assessed using western blotting. (C) The amount of IL-1β and lactate dehydrogenase (LDH) released into the culture supernatants of human peripheral neutrophils treated with MVs for 4 h (n=3 wells per group). (D) Representative immunostaining images for DNA (DAPI, blue), MPO (green), and GSDMD (red) in human peripheral neutrophils after exposure to MVs for 4 h in the presence of the ROS scavenger, MitoTempo (10 µM). Scale bar, 20 µm. (n=3 wells per group). (E) Representative Sytox Green fluorescence image for NETs formation after human peripheral neutrophils were stimulated with MVs for 4 h in the presence of MitoTempo (10 µM). Scale bar, 100 µm. (n=3 wells per group). (F) Mitochondrial membrane potential in human peripheral neutrophils was detected using flow cytometry, following exposed to MVs for 4 h in the presence of disulfiram (100 µM). Scale bar, 100 µm. (n=3 wells per group). Results are represented as mean ±SEM.

The caspase-11/GSDMD pathway contributes to NETs release and organ dysfunction in sepsis **28.** Herein, we explored whether the mtROS/GSDMD axis plays an important role in NETs formation during neutrophils treated with pyroptotic macrophage-derived MVs. Full-length gasdermin D (GSDMD) was downregulated while GSDMD-N was significantly upregulated in human neutrophils treated with macrophage-derived MVs, compared to that with the control macrophage-derived MVs or PBS **(Fig. 4A-B)**. GSDMD-N mediates the production and secretion of IL-1β 29; therefore, we evaluated the expression of IL-β in neutrophils. Cleaved IL-1β was significantly increased in the pyroptotic macrophage-derived MVs treated-neutrophils compared to that in neutrophils treated with control macrophage-derived MVs and PBS. This was accompanied by a significant production of lactate dehydrogenase (LDH) and IL-1β in cell culture supernatant **(Fig. 4C)**. Pyroptotic macrophage- derived MV exposure can increase the production of ROS in mitochondria. ROS production in mitochondria promotes gasdermin D oligomerization, pore formation, and pyroptosis in macrophages **30**. Therefore, we included MitoTEMPO, a superoxide scavenger, to the pyroptotic macrophage-derived MVs treatment. MitoTEMPO partially recovered the expression of full-length gasdermin D **(Fig. 4D)**. It reduced NETs formation in neutrophils exposed to pyroptotic macrophage-derived MVs **(Fig. 4E).** Therefore, mtROS could be upstream of the NETs formation pathway.

Disulfiram, a GSDMD inhibitor, inhibits NETs formation **28**. We evaluated whether disulfiram could reduce NETs formation in the presence of pyroptotic macrophage-derived MVs. Disulfiram reduced NETs formation when human neutrophils were exposed to pyroptotic macrophage-derived MVs **(Fig. 4F)**. We assessed whether DNase I could inhibit NETs formation induced by pyroptotic macrophage-derived MVs. DNase I was added to human neutrophils before pyroptotic macrophage-derived MVs treatment; DNase I reduced NETs formation, and the cells swelled and ruptured **(Fig. Supplemental 5A)**. Neutrophil elastase (NE) plays an important role in NETs formation **31**. We added sivelestat (NE inhibitor) to the neutrophils before exposure to pyroptotic macrophage-derived MVs. NE inhibitor did not inhibit NETs formation **(Fig. Supplemental 5B)**. Therefore, pyroptotic macrophage-derived MVs-induced NETs formation can be reversed by DNase I, and it occurs through a NE-independent pathway. The studies indicated that mtROS/GSDMD axis is involved in the macrophage pyroptosis**32** .To investigate whether inhibition of pyroptosis or mtROS/GSDMD axis leads to decrease of MVs release and in NETs formation, we have used the MCC950 (NLRP3 inhibitor), mitoTEMPO (a mitochondria-specific superoxide scavenger) or Disulfiram (GSDMD inhibitor) to treat macrophage. The results revealed that MCC950, mitoTEMPO or Disulfiram have significantly reduced MVs release **(Fig. Supplemental 6A)**. Next, we have performed the neutrophil co-culture with MVs derived from pyroptotic macrophage pretreated with DMSO, MCC950, mitoTEMPO or Disulfiram. The results showed that MCC950, mitoTEMPO or Disulfiram treated pyroptotic macrophages MVs have low activities to induced NETs formation compared with the pyroptotic macrophage MVs **(Fig. Supplemental 6B)**. Take together, these results indicated that pyroptotic macrophage-derived MVs regulated NETs formation by triggering the mtROS/ GSDMD axis.

### GSDMD-N-expressing mitochondria from pyroptotic macrophage-derived MVs contribute to NETs formation and mitochondrial dysfunction in neutrophils

**Figure 5:**
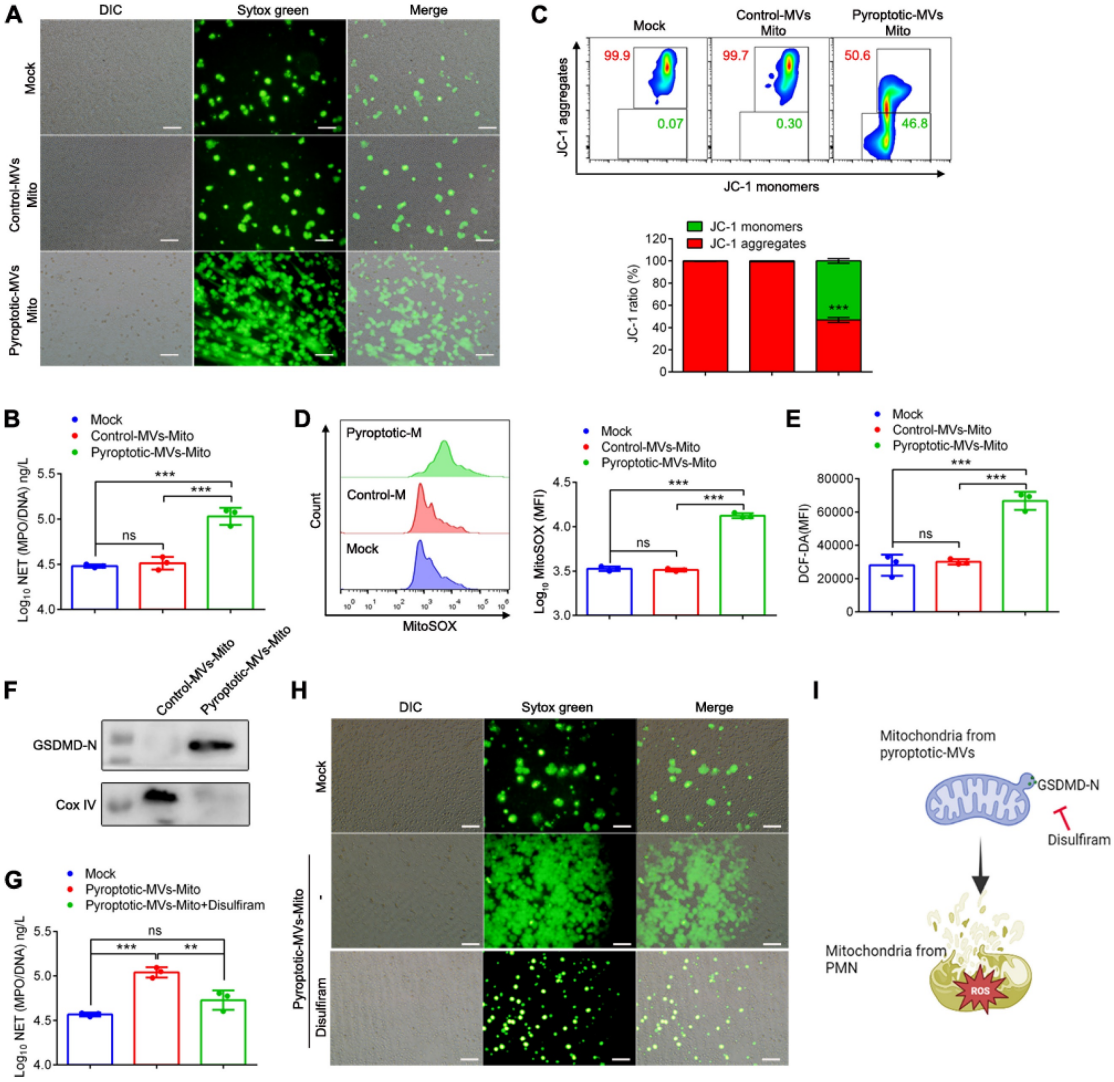
** GSDMD-N-expressing mitochondria from pyroptotic macrophage-derived MVs contribute to NETs formation and mitochondrial dysfunction in neutrophils.** Human peripheral neutrophils were isolated and cultured with PBS, mitochondria from control macrophage-derived MVs, or mitochondria from pyroptotic macrophage-derived MVs. (A) Representative Sytox Green fluorescence image for NETs formation after human peripheral neutrophils were stimulated with mitochondria for 4 h. Scale bar, 50 µm. (n=3 wells per group). (B) The concentration of MPO-DNA complexes released by human peripheral neutrophils assessed using ELISA. (n=3 wells per group). (C) Mitochondrial membrane potential in human peripheral neutrophils detected using flow cytometry after exposure to mitochondria for 1 h (n=3 wells per group). (D) MitoSOX fluorescence in human peripheral neutrophils measured using flow cytometry after exposure to mitochondria for 1 h. (n=3 wells per group). (E) Reactive oxygen species generated by human peripheral neutrophils were detected using 2,7,-dichlorofluorescein diacetate (DCF-DA) staining. (n=3 wells per group). (F) The expression of GSDMD-N in mitochondria from control macrophage-derived MVs and mitochondria from pyroptotic macrophage-derived MVs was assessed using western blotting. (G) The concentration of MPO-DNA complexes released by human peripheral neutrophils following treatment with mitochondria in the presence of disulfiram was assessed using ELISA. (n=3 wells per group). (H) Representative Sytox Green fluorescence image for NETs formation after human peripheral neutrophils were stimulated with mitochondria for 4 h in the presence of disulfiram (disulfiram (100 µM). Scale bar, 50 µm. (I) Schematic representation of the GSDMD-N-expressing mitochondria treated with disulfiram and then added into neutrophils. Results are represented as mean ±SEM.

Microvesicles from pyroptotic macrophages can induce NETs formation and mitochondrial dysfunction in neutrophils. In addition, these microvesicles contain mitochondria. To directly test the role of mitochondria in MVs, we extracted mitochondria from these MVs, and we added them to neutrophils to detect NETs formation and mitochondrial function in human neutrophils. Mitochondria extracted from pyroptotic macrophage-derived MVs induced marked NETs formation in human neutrophils **(Fig. 5A and B)**. In addition, the decreased mitochondrial membrane potential **(Fig. 5C)**, increased mtROS **(Fig. 5D)** and ROS **(Fig. 5E)** in human neutrophils collectively demonstrated that mitochondrial extracted from pyroptotic macrophage-derived MVs could be a significant factor contributing to NETs formation and mitochondrial dysfunction. We aimed to identify the factors on mitochondria that possessed the ability to induce NETs formation. Using western blotting, we confirmed that pyroptotic macrophage-derived MVs had considerably more GSDMD-N-expressing mitochondria compared to the control macrophage-derived MVs **(Fig. 5F).** Disulfiram can inhibit the function of GSDMD-N; therefore, we assessed whether the NETs induction ability of mitochondria extracted from the pyroptotic macrophage-derived MVs was attenuated following disulfiram treatment; these mitochondria did lose the ability to induce NETs formation **(Fig. 5G and H)**. Therefore, the expression of GSDMD-N on mitochondria of pyroptotic macrophage-derived MVs contributes to NETs formation and mitochondrial dysfunction, which can be inhibited by disulfiram.

### Pyroptotic macrophage-derived MVs induce tissues damage and coagulation when administered into mice

**Figure 6:**
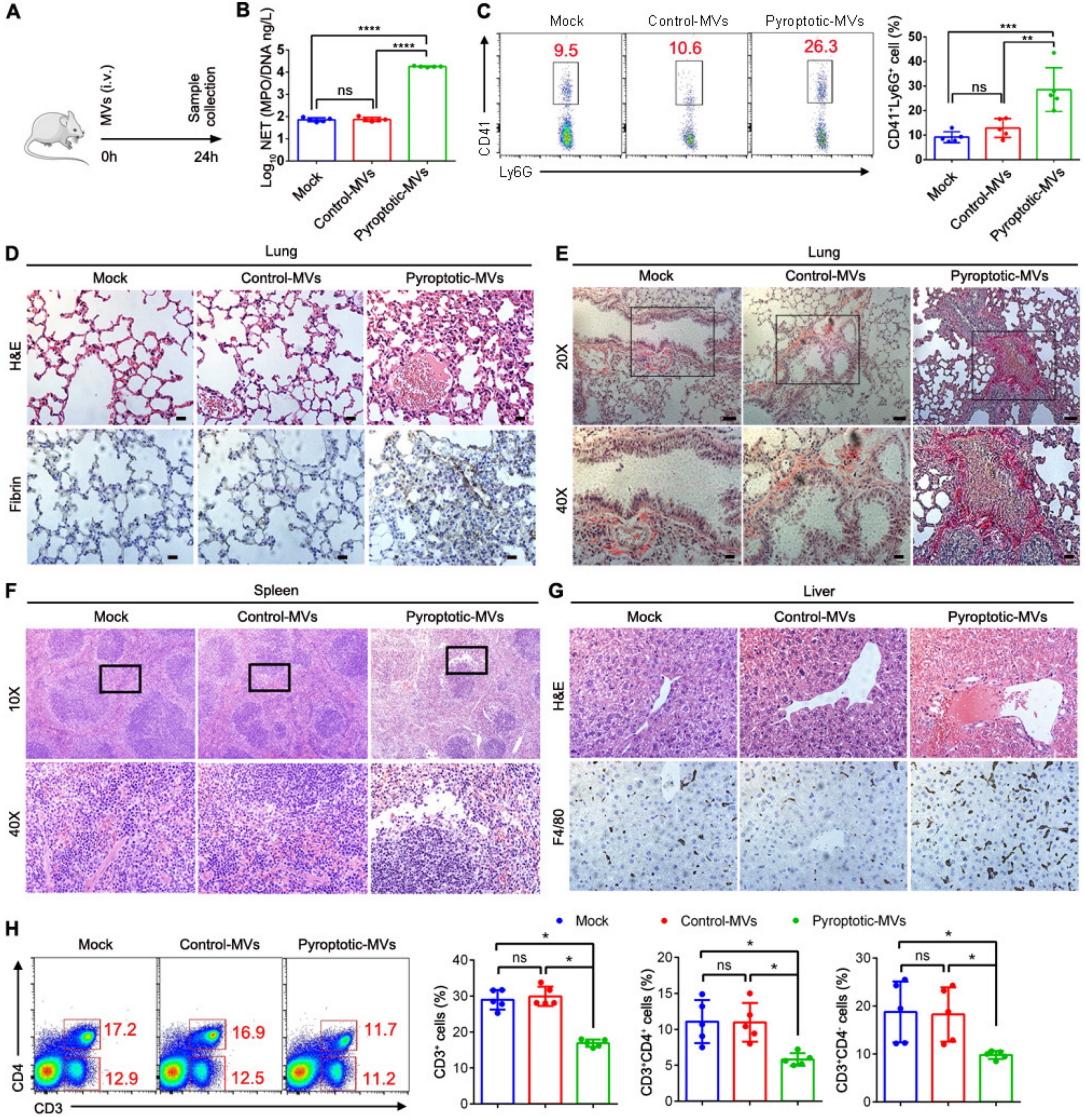
** Pyroptotic Macrophage-derived MVs Induces tissue Damage and Coagulation.** Schematic representation of mice administered intravenously with PBS, control macrophage-derived MVs, or pyroptotic macrophage-derived MVs for 24 h. (B) Circulating concentrations of MPO/DNA-NETs quantified using ELISA. (n=5 mice per group). (C) Blood platelet-neutrophil aggregates detected using flow cytometry. (n=5 mice per group). (D) Representative images of H&E staining of lung and lung sections stained with anti-fibrin. Scale bar, 20 µm (n=3 mice per group). (E) Representative images of Sirius red staining of lung. Scale bar, 20 µm (n=3 mice per group). (F) Representative histopathology images of spleen tissue section. Scale bar, 20 µm. (n=3 mice per group). (G) Representative histopathology images of liver and Fibrin in liver measured using immunohistochemistry. Scale bar, 20 µm. (n=3 mice per group). (H) The percentage of CD3^+^T cells and CD3^+^CD4^+^ T cells in spleen was detected using flow cytometry. (n=5 mice per group). Results are represented as mean ±SEM.

The potent ability of these MVs to induce NETs formation *in vitro* suggested that these MVs might disrupt the tissues and induce coagulation. To test this* in vivo*, MVs were administered into mice through tail vein injection (1×10^7^ MVs/mouse) **(Fig. 6A)**. We observed increased plasma levels of MPO/DNA in the pyroptotic macrophage-derived MVs treatment group but not in the control macrophage-derived MVs treatment group **(Fig. 6B)**. Platelet-neutrophil complexes play a critical pathophysiological role in facilitating NETs formation, and then favouring disseminated coagulation and organ failure in sepsis **33**. The blood Ly6G^+^ neutrophils in pyroptotic macrophage-derived MVs-treated mice had upregulated expression of the CD41^+^ platelet-specific marker, suggesting that the number of platelet-neutrophil complexes is higher in these mice than those treated with PBS or control macrophage-derived MVs **(Fig. 6C)**. Multi-organ failure is a proximal cause of death in sepsis**34**. Therefore, we assessed whether pyroptotic macrophage-derived MVs had an effect on organ dysfunction in sepsis. Not control macrophage-derived MVs but pyroptotic macrophage-derived MVs caused marked lung tissue damage, fibrin accumulation, and collagen deposition in the lung tissues compared to PBS **(Fig 6D-E).** In addition, pyroptotic macrophage-derived MVs induced marked spleen injury **(Fig. 6F)**. This was accompanied by significant reduction in the percentage of CD3^+^T cells, CD3^+^CD4^-^ T cells, and CD3^+^CD4^+^T cells in the spleen **(Fig. 6H)**. Not control macrophage-derived MVs but pyroptotic macrophage-derived MVs induced significant liver injury and fibrin accumulation in the liver, compared to PBS **(Fig. 6G)**. Therefore, pyroptotic macrophage-derived MVs play a critical role in the pathophysiology of sepsis.

### Disulfiram inhibits platelet activation and NETs formation during sepsis

**Figure 7:**
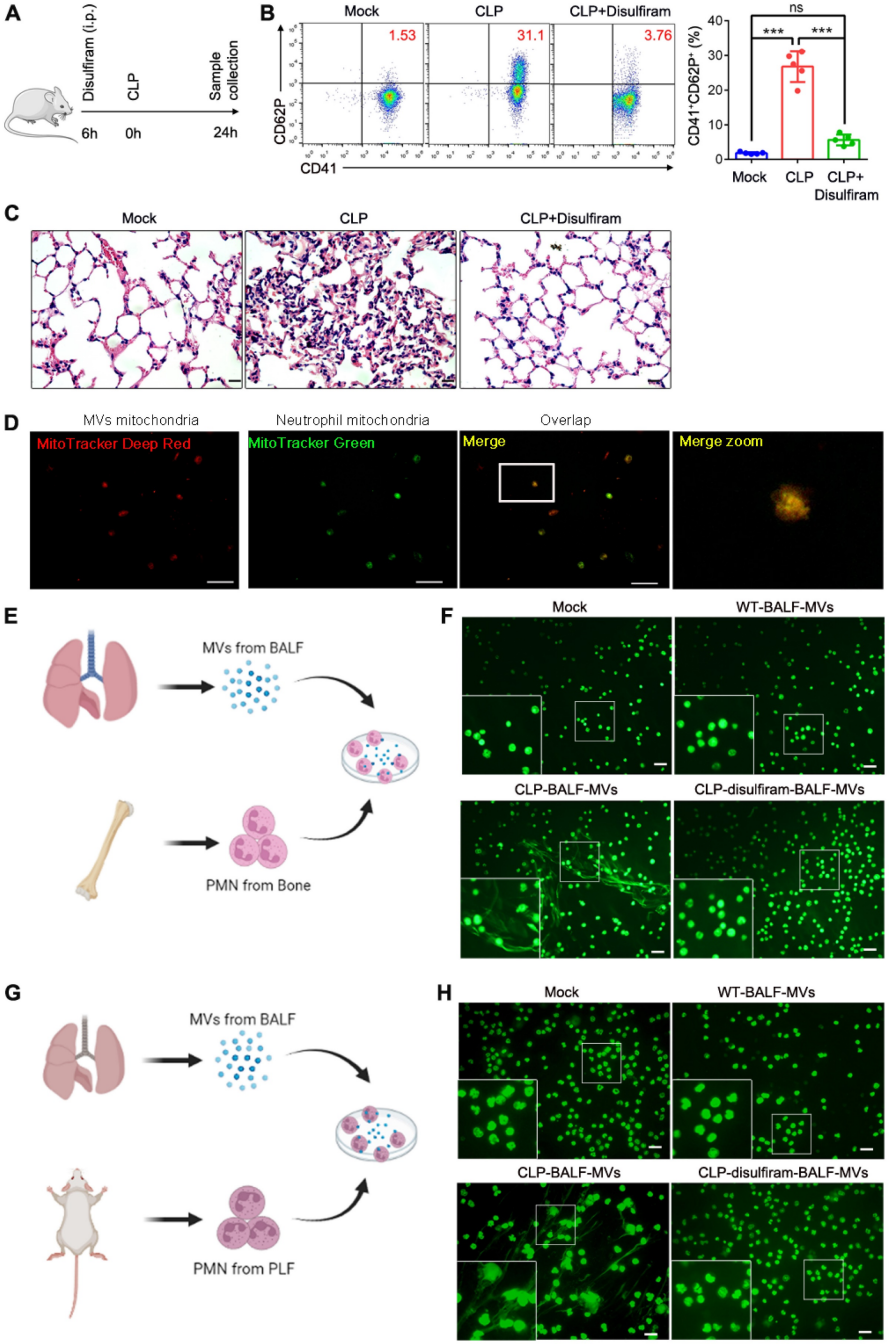
** Disulfiram Alleviated the Effect of MVs Obtained From BALF on NETs Formation during Sepsis.** Schematic representation of mice i.v. treated with disulfiram before the mice were subjected to CLP for 24 h. (B) The percentage of blood CD41+CD62P+ platelets was detected using flow cytometry (n=5 mice per group). (C) Representative images of H&E staining of lung tissue sections. Scale bar, 20 µm. (n=3 mice per group). (D) Representative fluorescent microscopy images of BALF-derived MV mitochondria internalization in neutrophils. Scale bar, 20 µm. (E) Experimental Schema. (F) BM neutrophils isolated from CLP-exposed mice were co-cultured with BALF-MVs from sham-operated group, sepsis mice, and disulfiram treatment group for 4 h. NETs were detected using Sytox Green fluorescence. Scale bar, 20 µm. (G) Experimental Schema. (H) Peritoneal neutrophils isolated from CLP-induced mice were co-cultured with BALF-MVs from sham-operated group, sepsis mice, and disulfiram treatment group for 4 h. NETs were detected using Sytox Green fluorescence. Scale bar, 20 µm. Results are represented as mean ±SEM.

NETs play a critical role in sepsis **35**. During sepsis process, MVs released by the cells can induce NETs formation. Activated platelets also play a vital role in sepsis by inducing NETs formation**36**. To determine the role for pyroptotic macrophage-derived MVs in inducing NETs formation, we used disulfiram (an inhibitor of pyroptosis) to treat sepsis mice **(Fig. 7A)**; the platelet activation was reduced, compared to that in CLP-stimulated mice **(Fig. 7B)**. Histological analysis showed that disulfiram-treated mice had reduced lung tissue oedema and vascular congestion compared to CLP-stimulated mice **(Fig. 7C)**. We further examined whether MVs isolated from BALF could induce NETs formation; mitochondria from BALF MVs could transfer to neutrophils **(Fig. 7D)**. To further confirm that MVs from disulfiram treated-CLP mice can reduce NETs formation, we cultured CLP mouse BM neutrophils or peritoneal neutrophils with MVs from normal mice, CLP mice, and disulfiram-treated CLP mice. Consistent with our previous findings, these MVs from CLP mice induced a higher NETs formation, when cultured with BM neutrophils from CLP mice. MVs from disulfiram treated-mice induced a lower NETs formation **(Fig. 7E and F).** Similar results were observed in peritoneal neutrophils **(Fig. 7G and H)**. Disulfiram can inhibit the ability of endogenous pyroptotic macrophage-derived MVs to induce NETs formation during sepsis.

### Disulfiram protects mice from acute lung injury (ALI) during LPS challenge

**Figure 8:**
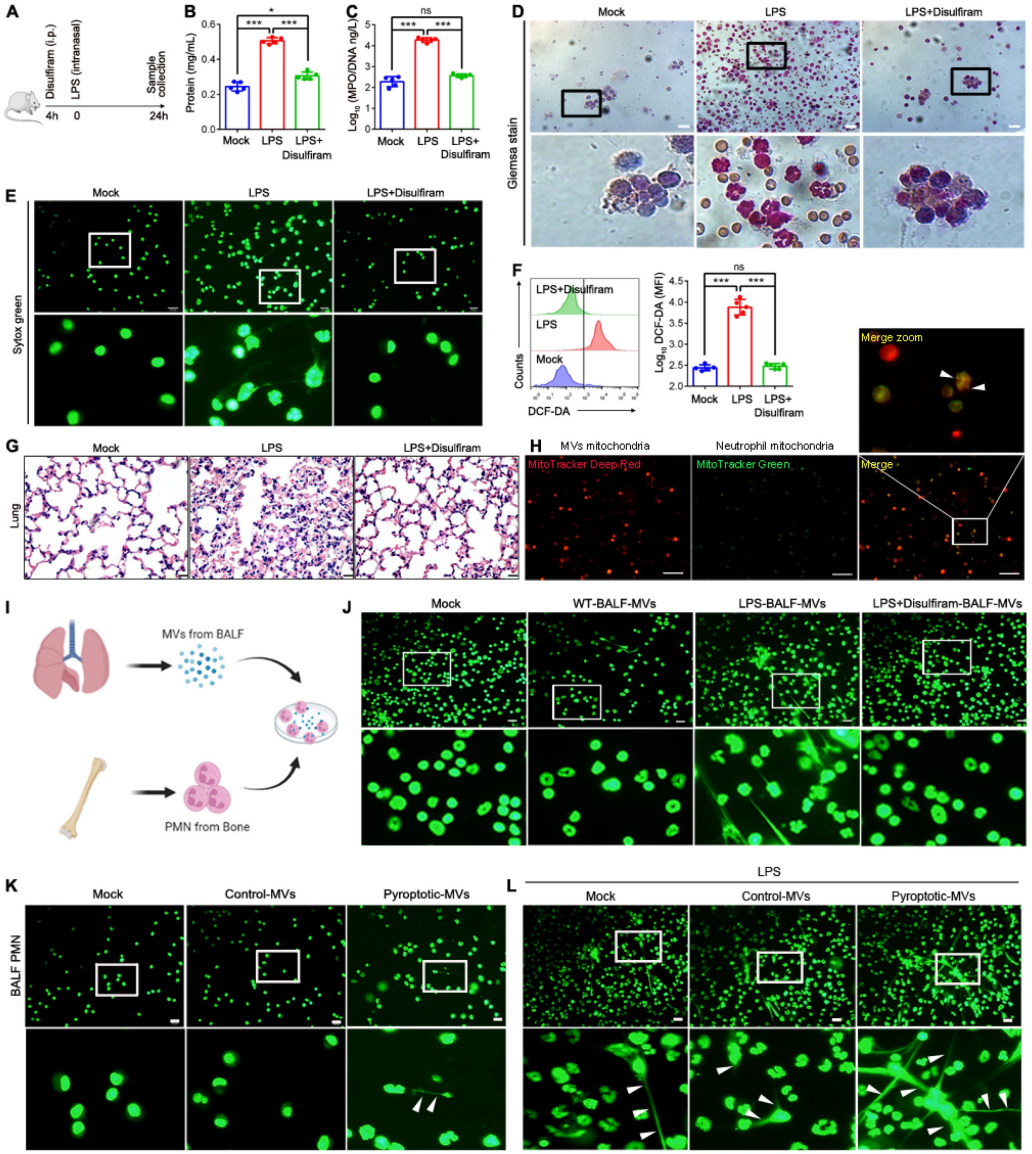
** Disulfiram Alleviated the Effect of MVs Obtained from BALF on NETs Formation during ALI Induction.** Schematic representation of mice treated with disulfiram for 4 h; the mice were then subjected to LPS treatment for 24 h. (B) The BALF supernatant was analysed with Coomassie Brilliant Blue G assay. (n=5 mice per group). (C) Concentrations of MPO/DNA-NETs in BALF were assessed using ELISA. (n=5 mice per group) (D) Representative images of Jimsa staining for inflammatory cell recruitment into the airspaces (original magnification, x20). (E) Representative fluorescent microscopy images of NETs formation in BALF. (n=5 mice per group). (E) Reactive oxygen species generation in BALF assessed using flow cytometry. (n=5 mice per group) (F) Quantification of reactive oxygen species generation in BALF. (n=5 mice per group) (G) Representative images of haematoxylin and eosin-stained lung tissue from mice following LPS induction for 24 h. (n=3 mice per group). (H) Representative fluorescent microscopy images of BALF-derived MV mitochondria internalization in neutrophils. Scale bar, 20 µm. (I) Schematic representation of MV isolation from BALF; they were then added into BM neutrophils. (J) Fluorescence microscopy images showing NETs formation from representative ALI neutrophils following incubation with BALF-MVs for 4 h. Neutrophil DNA stained with Sytox Green. (K) Administration of ectogenic pyroptotic macrophage-derived MVs induced more NETs in BALF. Neutrophil DNA was stained with Sytox Green. (L) Administration of ectogenic pyroptotic macrophage-derived MVs induced more NETs in the BALF of mice with LPI-induced ALI. Neutrophil DNA was stained with Sytox Green. Results are represented as mean ±SEM.

Sepsis can induce ALI, and NETs formation contributes to ALI**37, 38**. Macrophage pyroptosis is observed in ALI**39-41.** To assess the potential therapeutic value of disulfiram in an ALI model, we evaluated whether disulfiram administration prevents ALI in a murine model by reducing NETs formation. Mice were treated with PBS or disulfiram for 4 h, and were then challenged with LPS; the levels of NETs, ROS generation, and organ dysfunction were evaluated after 24 h **(Fig. 8A)**. Disulfiram reduced lung vascular permeability, neutrophil infiltration, NETs formation, and ROS generation in BALF in mice exposed to LPS **(Fig. 8B-F)**. Histological analysis showed reduced lung injury in mice pre-treated with disulfiram even after LPS inhalation **(Fig. 8G)**. Therefore, disulfiram can protect mice from ALI during LPS challenge by inhibiting NETs formation and ROS generation. We further examined whether MVs isolated from the BALF of disulfiram-treated mice could induce NETs formation. We found that mitochondria could transfer to neutrophils through MVs **(Fig. 8H)**. The MVs from the disulfiram-treated ALI group could not induce NETs formation when co-cultured with bone marrow-derived neutrophils *in vitro*; this was in contrast to the results for MVs from mice treated with LPS **(Fig. 8I and J)**. We evaluated the effect of exogenous pyroptotic macrophage-derived MVs on NETs formation* in vivo* through intranasal instillation. Pyroptotic macrophage-derived MVs, but not control macrophage-derived MVs, induced more neutrophils and NETs formation in the BALF, compared to that in the treatment with PBS **(Fig. 8K)**. Treatment of mice with pyroptotic macrophage-derived MVs 24 h after LPS challenge induced more NETs formation; this effect was not seen in the control macrophage-derived MVs-treated mice **(Fig. 8L)**. Therefore, pyroptotic macrophage MVs from BALF of ALI model can induce NETs formation, which can be partly inhibited by disulfiram.

## Discussion

NETs not only function to protect their host from infection but also can drive the pathology in many diseases. However, the molecular mechanism of NETs formation during sepsis needs to be better understood. Here we identify pyroptotic macrophage-derived MVs that can transfer mitochondria to neutrophils and induce NETs formation and ROS production. The mtROS/gasdermin D axis could contribute to NETs formation in response to pyroptotic macrophage-derived MVs. Interestingly, GSDMD-N-expressing mitochondria from pyroptotic macrophage-derived MVs contribute to NETs formation and mitochondrial dysfunction in neutrophils. In addition, pyroptotic macrophage-derived MVs induced organ dysfunction and systemic coagulation *in vivo*. However, following treatment with disulfiram, the effect on NETs formation was alleviated. In this study, the interaction between macrophages and neutrophils was elucidated, and it provides a new pathway and therapeutic target for NETs formation during sepsis.

Extracellular vesicles contribute to NETs formation, especially those from platelets. Jiao et al., **20** showed that there is a reduction in NETs formation during sepsis when platelets are depleted. The Akt/mTOR pathway induces NETs formation through exosomal high-mobility group protein 1 (HMGB1), miR-15b-5p, and miR-378a-3p; dengue virus (DV) activates platelets through CLEC2 to release EVs and further activate CLEC5A to induce NETs formation**42**. Maugeri et al., demonstrated that microparticles released from activated platelets in the blood during systemic sclerosis interacted with neutrophils, promoted neutrophil autophagy, and generated NETs**43.** However, whether pyroptotic macrophage-derived MVs contribute to NETs formation and the underlying mechanism was unknown. Here, we demonstrated that they serve as a new fundamental checkpoint that can be used to regulate NETs formation during sepsis.

Pyroptotic macrophage-derived MVs enhance NETs formation; therefore, we aimed to elucidate the mechanism that which functional mitochondria of the pyroptotic macrophages taken up by neutrophils could modulate NETs formation. Mitochondrial dysfunction contributes to the development in various forms of severe illness**44, 45**. Pyroptotic macrophages transferred mitochondria to neutrophils through MVs internalization. The mitochondrial membrane potential and mitochondria distribution decreased significantly. There was an increase in the production of mtROS, which contributed to the progression of mitochondrial dysfunction. This dysfunction leads to a breakdown of mitochondrial membrane integrity, the ablation of ΔΨ, and ultimately damages the cells further**46-48**. These data were consistent with the results reported by Silva et al.; **22** MSCs could deliver their mitochondria to alveolar epithelial and endothelial cells through extracellular vesicles. This transfer rescues mitochondrial membrane potential and reduces the production of mtROS. Adding mitochondria from pyroptotic macrophage-derived MVs to neutrophils induced NETs formation and neutrophil mitochondrial dysfunction. However, when these GSDMD-N-expressing mitochondria were treated with disulfiram, the NETs induction ability was attenuated. Therefore, GSDMD-N-expressing mitochondria are the main regulators in pyroptotic macrophage-derived MVs, which contribute to neutrophil mitochondria dysfunction and NETs formation during sepsis. These results are supported by earlier ones that show that GSDMD-N domains bind membrane lipids and form membrane pores **49**, and that disulfiram targets cys191 on human GSDMD to inhibit pore formation **50**. We aimed to elucidate the downstream signalling pathway involved in modulating NETs formation through pyroptotic macrophage-derived MVs. GSDMD regulates NETs formation **28**. RagA and RagC increase the production of mtROS, which accelerates the oligomerization of GSDMD in macrophages. Therefore, mtROS might contribute to the formation of plasma membrane pores through activating GSDMD in macrophages **30**, raising the possibility that neutrophils might implement a similar mechanism. The production of mtROS following mitochondrial transfer resulted in GSDMD cleavage and induction of NETs formation. When mtROS production was inhibited using MitoTempo, a mitochondria-specific superoxide scavenger, the expression of GSDMD was recovered. Therefore, mtROS is upstream of GSDMD. Several pathways are involved in NETs formation; NE is one pathway involved in chromatin decondensation and histone degradation modulated by ROS-dependent translocation from granules into nucleus **31**. Another pathway involves the activation of the enzyme, arginine deiminase 4 (PAD4), which is triggered by a spike in cytosolic calcium. This pathway promotes chromatin decondensation through histone citrullination **51**. Non-canonical inflammasome signalling induces the formation of gasdermin D-dependent NETs **52.** LPS binds and triggers caspase-4/11 within the non-canonical inflammasome complex, and then the GSDMD pores in the nuclear membrane allow caspase-11 access to chromatin, mediating NETs formation. Sivelestat did not reduce NETs formation in the presence of pyroptotic macrophage-derived MVs, indicating that NETs formation involves the GSDMD-dependent pathway but not the NE-dependent pathway. Interestingly, there are other signaling pathways involved in NETs formation, such as direct protein kinase (PKC)/NADPH pathway^**53**^ and extracellular signal-regulated kinase (ERK) activation pathway**54**. Of note, someone demonstrates that NETs formation as a result of either pathway of inflammation activation did not require GSDMD**55**. These pathways may influence our observed results and needed to be further study in future.

Sepsis is characterized by inflammation, organ dysfunction, and coagulation disorders. To confirm the critical role of pyroptotic macrophage-derived MVs *in vivo*, mice were treated with pyroptotic macrophage-derived MVs or control macrophage MVs. Pyroptotic macrophage-derived MVs can induce platelet activation, platelet-neutrophil adherence, and fibrin in the liver and lungs; these contribute to coagulation. Pyroptotic macrophages release TF-positive MVs that can induce coagulation **19**. Hepatocyte pyroptosis results in the release of inflammasome particles, which induce stellate cell activation and liver fibrosis **56.** Pyroptotic macrophage-derived MVs induced lung collagen accumulation. In addition, they reduced the number of CD4^+^, CD8^+^, and CD3^+^ T cells in the spleen, which is characteristic of immunosuppression during sepsis **57**.

Disulfiram is used to treat chronic alcohol addiction **58**. It inhibits liposome leakage through inhibiting the GSDMD-mediated pore formation **50**. In addition, disulfiram reduces NETs formation, the processing of histone H3, and the level of organ injury markers; this improves the sepsis outcomes **28.** In this study, disulfiram not only inhibited NETs formation *in vitro* in the presence of pyroptotic macrophage-derived MVs, but also alleviated inflammation, NETs formation, and organic damage *in vivo* in a mouse model of sepsis and ALI. In addition, when the MVs from the BALF of disulfiram-treated mice were cultured with neutrophils from the CLP peritoneal lavage fluid and bone marrow, NETs formation was reduced compared to that in MVs from PBS-treated mice. MVs isolated from the BALF of ARDS model mice induced NETs formation; this was not the case with MVs from control mice. When mice were pre-treated with disulfiram, these effects were alleviated. BALF from ARDS patients induces NETs formation, but not control BALF **59**. Disulfiram inhibits NETs formation and protects rodents from ALI induced by SARS-CoV-2 **60**. The pyroptotic bodies released in the early phase of ALI promote activation of epithelial cells and recruit neutrophils **39.** In addition, these MVs induce NETs formation, which in turn contributes to macrophage pyroptosis **61**, forming a positive feedback loop that exacerbates organ dysfunction and systemic coagulation in sepsis.

We elucidated that MVs from pyroptotic macrophages interacted and induced neutrophil activation and NETs formation during sepsis. However, many components, such as platelets, neutrophils, and erythrocytes, play an important role in the pathogenesis of sepsis. They release microvesicles to regulate the prognosis of sepsis. The red blood cell MVs activate both FXII and prekallikrein to mediate inflammatory and thrombotic outcomes^62^. In addition, once activated, platelets release extracellular vesicles (EVs), which contain proteasomes. These proteasomes process exogenous ovalbumin (OVA) and load their antigenic peptide into MHC-I molecules, which promote OVA-specific CD8^+^ T-lymphocyte proliferation^63^. Furthermore, exosomal prostagland in E2 (PGE2) from M2 macrophages inhibits NET formation through lipid mediator class switching in sepsis. Interestingly, extracellular vesicles derived from mesenchymal stromal cells modulate the formation of neutrophil extracellular traps by transferring mitochondria. We cannot prove that these MVs from pyroptotic macrophage are the only factor affecting NET formation during sepsis, but they are definitely the main factor. Future studies should focus on elucidating whether the function of these MVs mediate sepsis development and whether these microvesicles from different cells interact with each other. MVs are internalized by immune and non-immune cells involved in sepsis development; this needs further studies. We demonstrated a pathway for NETs formation following the challenge with pyroptotic macrophage-derived MVs; there could be other pathways that contribute to this. Several mechanisms of mitochondrial transfer, such as endocytosis and connexin43-dependent mechanisms, have been identified **64.** However, these mechanisms were not investigated in this study and may need further investigation.

This is the first study to assess the ability of pyroptotic macrophage-derived MVs to mediate NETs formation by transferring GSDMD-N-expressing mitochondria and triggering the mtROS/GSDMD axis, which contributes to organ dysfunction and systemic coagulation during sepsis. Therefore, this study offers new perspectives into the role of GSDMD-N-expressing mitochondria during sepsis pathogenesis.

## Materials and methods

### Ethics statement

C57BL/6 (6-8 weeks old) male mice were housed in suitable conditions under the supervision of the Fifth Affiliated Hospital of Sun Yat-Sen University. The Ethics Committee Board for Human Experiments at Fifth Affiliated Hospital of Sun Yat-Sen University approved this study and all experimental protocols (approval number 00263). Healthy people gave informed consent to all experiments.

### Cecal Ligation and Puncture model

The mice model of sepsis was induced through cecal-ligation and puncture (CLP), as described previously **28.** Mice were injected intraperitoneally with either PBS or disulfiram (80 mg/kg, n=5/ per group) 4 h before being challenged with CLP.

### Mice Inhaled-LPS model

Mice inhaled-LPS model was developed as described previously **22**. Briefly, mice were intraperitoneally injected with either PBS or disulfiram (240 mg/kg, n=5/ per group) 4 h before exposure to LPS (2 mg/kg in 50ul PBS) via the intranasal route. After 24 h, the lung was saved for histological analysis and BALF was harvested for further analysis.

## Experimental Model and Subject Details

### BMDM Cultures and stimulation

1 × 10^6^ BMDMS were seeded into 6 wells of RPMI-1640 medium with 15% L929-cell conditioned medium (LCM) and cultured for one week. To induce mouse BMDM pyroptosis, these cells were exposed to LPS (500 ng/mL) for 4-5 h, and then the media was discarded. Fresh media with nigericin was added, the cells were incubated for 2 h; the culture supernatant was collected for isolating microvesicles. To obtain control macrophage-derived MVs, BMDM were cultured with DMEM without FBS for 6-7 h; the culture supernatant was obtained for isolating microvesicles.

### THP-1 Cultures and stimulation

RPMI 1640 containing 10% FBS was used to culture THP-1 cells, and PMA at 5 ng/ml was used to induce macrophage differentiation. The cells were then treated with fresh media containing nigericin for 90 min; the culture supernatant was collected for isolating microvesicles. To obtain control macrophage-derived MVs, following THP1 treatment, the cells were treated with PMA; the media was diacarded, and the cells were cultured with DMEM without FBS for 3-4 h. The culture supernatant was obtained for isolating microvesicles.

### Murine platelet isolation

Following anaesthesia with isopentane, murine blood was collected through cardiac puncture. Platelet-rich plasma (PRP) was acquired from whole blood through centrifugation at 100x g for 10 min without brake, as described previously **65**. The platelets were washed with PBS, and collected through centrifugation at 2000× *g* for 10 min without brake.

### Neutrophil isolation and stimulation

Human circulating neutrophils and mouse bone marrow neutrophils were isolated using a neutrophil isolation kit. In addition, to investigate the effect of MVs on neutrophils, we isolated mouse neutrophils from peritoneal lavage fluids of the CLP model by magnetic bead-based separation method (Stemcell, #19762). To detect the effect of pyroptotic macrophage-derived MVs on NETs formation, human neutrophils were pre-exposed to disulfiram (GSDMD inhibitor, 120 μM), sivelestat (neutrophil elastase inhibitor, 10 μM), and mitoTEMPO (a mitochondria-specific superoxide scavenger, 10 μM) 1 h before stimulation, as described above.

### MVs isolation from cell culture supernatant and alveolar lavage fluid

We obtained MVs from cell culture supernatant and alveolar lavage fluid as described previously **66**. Cell debris was discarded from the cell culture supernatant following the various stimulations. All samples were centrifuged at 2600 ×*g* for 5 min to remove the cellular debris. MVs were obtained through centrifugation at 20,000 × *g* for 20 min at 4 °C. To obtain MVs from murine bronchoalveolar lavage, anesthetised mice were cut open, the lungs and trachea were exposed, and an 18-gauge venous catheter was inserted into the trachea. BALF was extracted by gently instilling 0.8 ml of PBS two times, and the MVs in BALF were isolated as described above.

### Mitochondrial isolation and quantification

A mitochondria isolation kit (Thermo Scientific, Cat# 89874) was used to isolate mitochondria from MVs, according to the manufacturer's instructions and the protocol used in a previous study **23**. Mitochondrial proteins were detected using the bicinchoninic acid (BCA) method. The equivalent of 0.2 µg of proteins was used to treat 1×10^5^ neutrophils for 2-3 h.

### Nanoparticle tracking analysis (NTA)

NanoSight NS 5330 was used to detect the size distribution and concentration of macrophage-derived MVs. Following the isolation of MVs from 1×10^6^ THP1 and BMDM cells, all samples were diluted in 1 mL distilled water. The detection threshold was set to detect as many particles as possible.

### Transmission electron microscopy (TEM)

Purified microvesicles were diluted with PBS and were analysed with ZetaView (Germany).

### Mitochondrial transfer from pyroptotic macrophage-derived MVs to neutrophils

Mitochondrial transfer was performed as described previously **22**. Briefly, THP1 cells, after LPS activation for 4 h, were washed with PBS; they were treated with MitoTracker Deep Red FM (200 nM) in the dark for 45 min. Cells were washed 5 times with PBS and then treated with nigericin (MCE) for 2 h to generate microvesicles. Neutrophils were seeded on coverslips pretreated with polylysine at a density of 1×10^5^ in a 24-well plate. After 2-3 h, neutrophils were washed with PBS and stained for endogenous mitochondria through incubation with MitoTracker Green (200 nM) for 45 min in the dark. Neutrophils were washed 5 times with PBS, and microvesicles were added for 1 h of co-incubation. The co-localization of endogenous mitochondria and the introduced mitochondria was observed using immunofluorescence microscopy.

### Assessment of mitochondrial membrane potential

The mitochondrial membrane potential of neutrophils was evaluated as previously described**22**. Briefly, neutrophils were incubated with PBS, control macrophage-derived MVs, and pyroptotic macrophage-derived MVs in a 24-well plate containing coverslips for 4 h. JC-1dye was added into the medium and incubated for 15 min at 37° C. In mitochondria, the dye accumulation is based on the potential of the mitochondrial membrane. Following staining, EVOS FL Auto epifluorescent microscope was used to image live cells at 40× magnification. Flow cytometry was used to detect mitochondrial membrane potential.

### Measurement of mitochondrial superoxide production

Mitochondrial superoxide production in neutrophils was evaluated as described previously **22**. Briefly, 96-well black plate with a clear bottom was seeded with 1x10^5^ neutrophils in triplicate under normal cell culture conditions. Cells were stimulated with PBS, control macrophage-derived MVs, and pyroptotic macrophage-derived MVs at 37°C for 4 h. After 20 minutes of incubation with MitoSOX (ThermoFisher Scientific, UK, 5μM) at 37° C, cells were washed using PBS. EVOS FL Auto epifluorescent microscope was used to image live cells at 20× magnification. Flow cytometry was used to detect MitoSOX.

### Western blotting analysis

Neutrophils were obtained from healthy people and incubated with PBS, control macrophage-derived MVs, and pyroptotic macrophage-derived MVs for 2 h. Cells were collected and lysed with RIPA buffer in the presence of protease inhibitor (Sigma Aldrich) and phenylmethylsulfonyl fluoride (PMSF) for 30 min on ice. BCA protein assay (Micro BCA protein assay kit) was used to assess the protein concentration according to the manufacturer's instructions. The same amounts of protein were subjected to 10% SDS-PAGE under reducing conditions and transferred onto a PVDF membrane (GE Healthcare, Germany). The membranes were incubated overnight with anti-GSDMD,** anti-IL-1β,** anti-GSDMD-N, or anti-GAPDH antibodies at 4° C after blocking with 5% milk. After washing, the blots were incubated with secondary antibodies for 2 h at 37° C. Protein expression was visualized on GE ImageQuant LAS 500 using an ECL kit (Fdbio SCience).

### Determination of neutrophil pyroptosis by lactate dehydrogenase (LDH) assay

The levels of LDH were measured using Roche's Cytotoxicity Detection Kit (Roche). Briefly, cell supernatants were centrifuged at 1000 ×*g* for 10 min at 4°C to remove cell debris; 50 µl of supernatant samples were placed in a 96-well plate. They were treated with 50 µl of the reaction mixture and mixed on a plate shaker for 1 min followed by incubation in the dark at room temperature for 30 min. The positive control was prepared by lysing cells with 2% Triton X-100 (Sigma Aldrich) for 10 min before collecting supernatants. FLUOstar Omega microplate reader was used to detect the optical densities at 405 nm. Results are presented as a % relative to the positive control.

### Flow cytometry

Anti-mouse CD41 (BioLegend, Cat#133913); anti-mouse CD62P (BioLegend, Cat#148305); anti-mouse Ly6G (BioLegend, Cat#127653) were used for flow cytometric analysis. Data were obtained on an LSRII (BD Biosciences) and analysed with FlowJo v10.07.

### Immunofluorescence

Neutrophils were seeded on coverslips pretreated with polylysine. Following the treatment with PBS, control macrophage-derived MVs, or pyroptotic macrophage-derived MVs, the cells were fixed in 4% paraformaldehyde for 10 min at room temperature (RT), and washed using PBS. Cells were permeabilized using 0.2% Triton X-100 for 5 min, and washed with PBS. Cells were blocked with 1% BSA for 30 min and stained with rabbit anti-histone H3 antibody (Abcam; 1:500); mouse anti-myeloperoxidase antibody (Abcam 1:500); rabbit anti-GSDMD (Abcam; 1:500); rabbit anti-TOM20 antibody (Abcam 1:500); mouse anti-8-OHdG antibody (Novus; 1:200). The samples were then incubated with Alexa Fluor 488 (1:1000, Thermo Fisher Scientific, USA) or Alexa Fluor 594 (1:1000, Thermo Fisher Scientific, USA) at RT for 1 h. DAPI (Solarbio, China) was used to stain the nuclei. Images were acquired using Axio Observer combined with fluorescence microscope system at 40×. All acquired images were analysed using ImageJ. Images were acquired using the microscope's associated software.

### Neutrophil extracellular traps (NETs) assays

To quantify NETs formation in the culture supernatant, mouse bone marrow neutrophils and human circulating neutrophils were seeded at a density of 1×10^5^ in a 96-well black plate. After treatment with MVs for 4 h, the supernatant was centrifuged at 2000-3000 ×*g* for 20 min, and the NETs in the supernatant were collected. In addition, the serum and BALF of mice were collected. All samples were tested using the MPO-DNA assay with a FlexStation 3 Microplate Reader.

### ROS measurement

Following the treatment of neutrophils with PBS, control macrophage-derived MVs, and pyroptotic macrophage-derived MVs, the cells were washed with PBS three times and resuspended in RPMI 1640. The fluorescent probe, 2'7'-dichlorofluorescein diacetate was added to the cell suspension and incubated at 37°C for 30 min. Flow cytometry was used to test ROS production.

### Cytokine assays (ELISA)

According to the manufacturer's instructions, ELISA was used to measure the IL-1β concentrations and NETs formation in the plasma and cell culture supernatant. The optical density of the sample at 450 nm was measured using a spectrophotometer (Spectra Max-250; Molecular Devices, Sunnyvale, CA, 302 USA).

### Histological and immunohistochemical examination

Mice were sacrificed 24 h after treatment. Tissues were collected, fixed with 4% buffered formalin, and embedded in paraffin blocks. A 5 mm section was stained with haematoxylin and eosin for histological analysis. Anti-fibrin at 4 μg/ml was used for staining fibrin deposition, with biotinylated goat anti-mouse IgG (Secondary antibody). Images were acquired using a DMI 6000B microscope (Leica Microsystems) at 200× or 400× magnification.

### Statistical analysis

Analyses were performed using GraphPad Prism 5 software. Means of three technical replicates were obtained as individual data points for each donor and pooled for statistical analysis. Summary data are presented as mean ±SEM. Post hoc analyses were performed on parametric data using Student's *t*-test or one-way ANOVA (Bonferroni's choice comparison). For non-parametric data, the Kruskal-Wallis test with post-hoc analysis using Dunn's selected comparisons was used. Statistical significance was set at p<0.05.

## Supplementary Material

Supplementary figures.

Supplementary movie.
