# Supplementary material for: Pyroptotic Macrophage-Derived Microvesicles Accelerate Formation of Neutrophil Extracellular Traps via GSDMD-N-expressing Mitochondrial Transfer during Sepsis
Source: Int J Biol Sci. 2024 Jan 1;20(2):733–50. doi: 10.7150/ijbs.87646 (PMC10758106; doi:10.7150/ijbs.87646)
Supplement: Supplementary file 1 — Supplementary figures. [file ijbsv20p0733s1.pdf]

Supplementary Materials

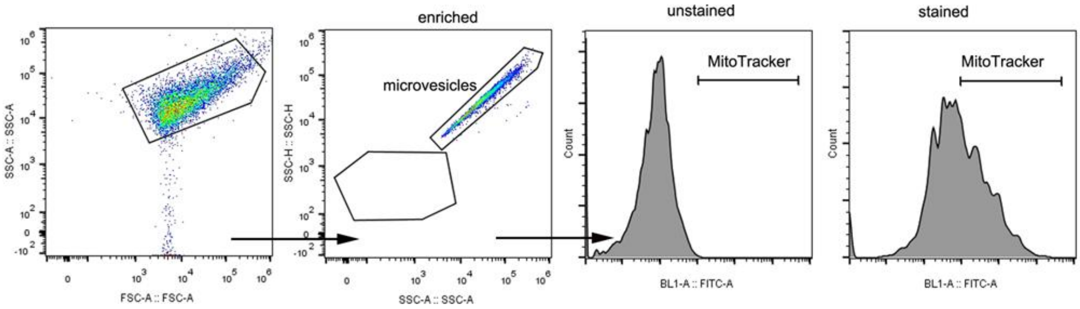

Fig S1. FACS plots of gating strategy used to identify sorting microvesicles with mitochondria. (Left plot) Standards defining size exclusion gate y axis side scatter (SSC-A) x axis forward scatter (FSC-A). Within the size exclusion gate, Crude and pure mitochondrial populations (Middle plot). MitoTracker green (MTG) (Right plot 100 nM) staining to identify mitochondrial percentage (Right plot).

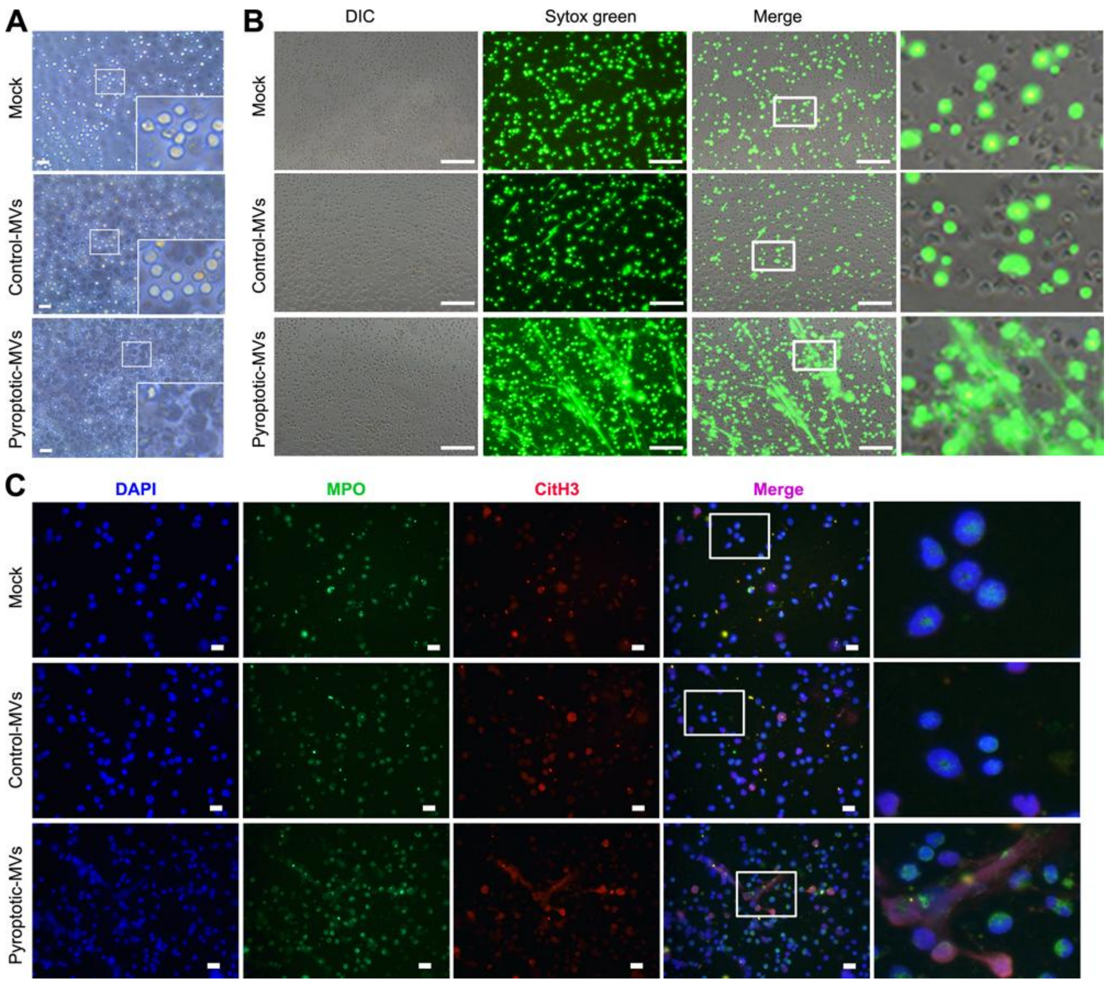

Fig. S2. Pyroptotic Macrophage-derived MVs induce mouse NET formation.

Mouse neutrophils were isolated from bone marrow and cultured with PBS, control macrophage-derived MVs or pyroptotic macrophage-derived MVs for 4 h at 37°C. **(A)** Morphology of mouse bone marrow neutrophils. Scale bar, 20μm. **(B)** Representative Sytox green fluorescence image for NETs formation of mouse bone marrow neutrophils. Scale bar, 50 µm. **(C)** Representative images showing neutrophil staining of DNA (DAPI, blue), myeloperoxidase (MPO, green), and the citH3 (red) of mouse bone marrow neutrophils. Scale bar, 20 µm.

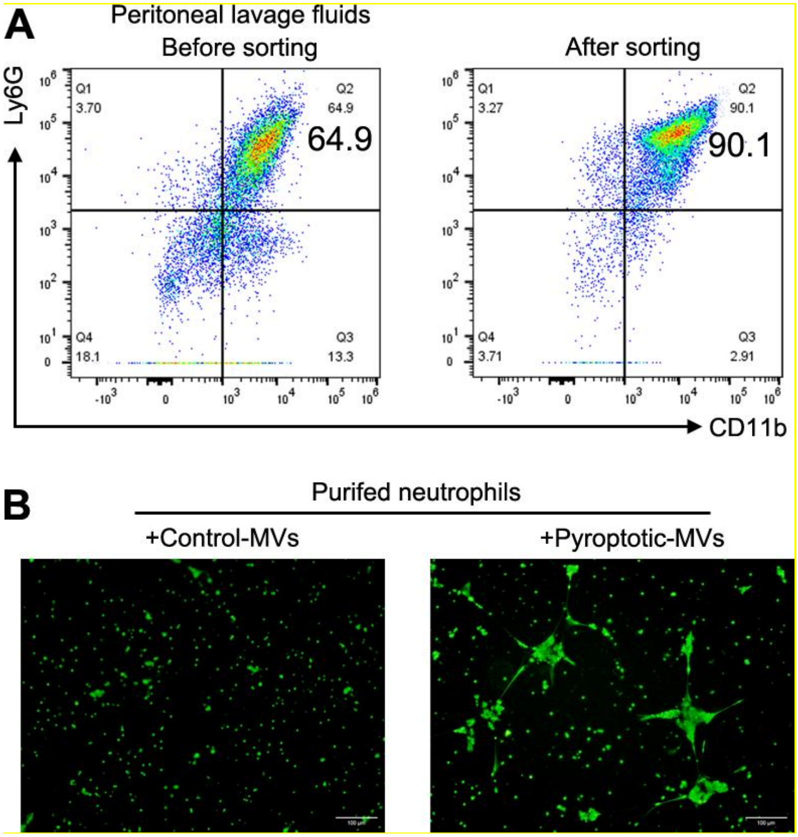

Fig. S3. Pyroptotic macrophage-derived MVs induce NETs formation.

Mouse neutrophils from peritoneal lavage fluids were sorted by magnetic bead-based separation method, and cultured with control macrophage-derived MVs, or pyroptotic macrophage-derived MVs for 4 h at 37°C. **(A)** The purify of sorting neutrophils was determined by flow cytometry. **(B)** Representative Sytox Green fluorescence image for NETs formation of human peripheral neutrophils. (n=3 wells per group).

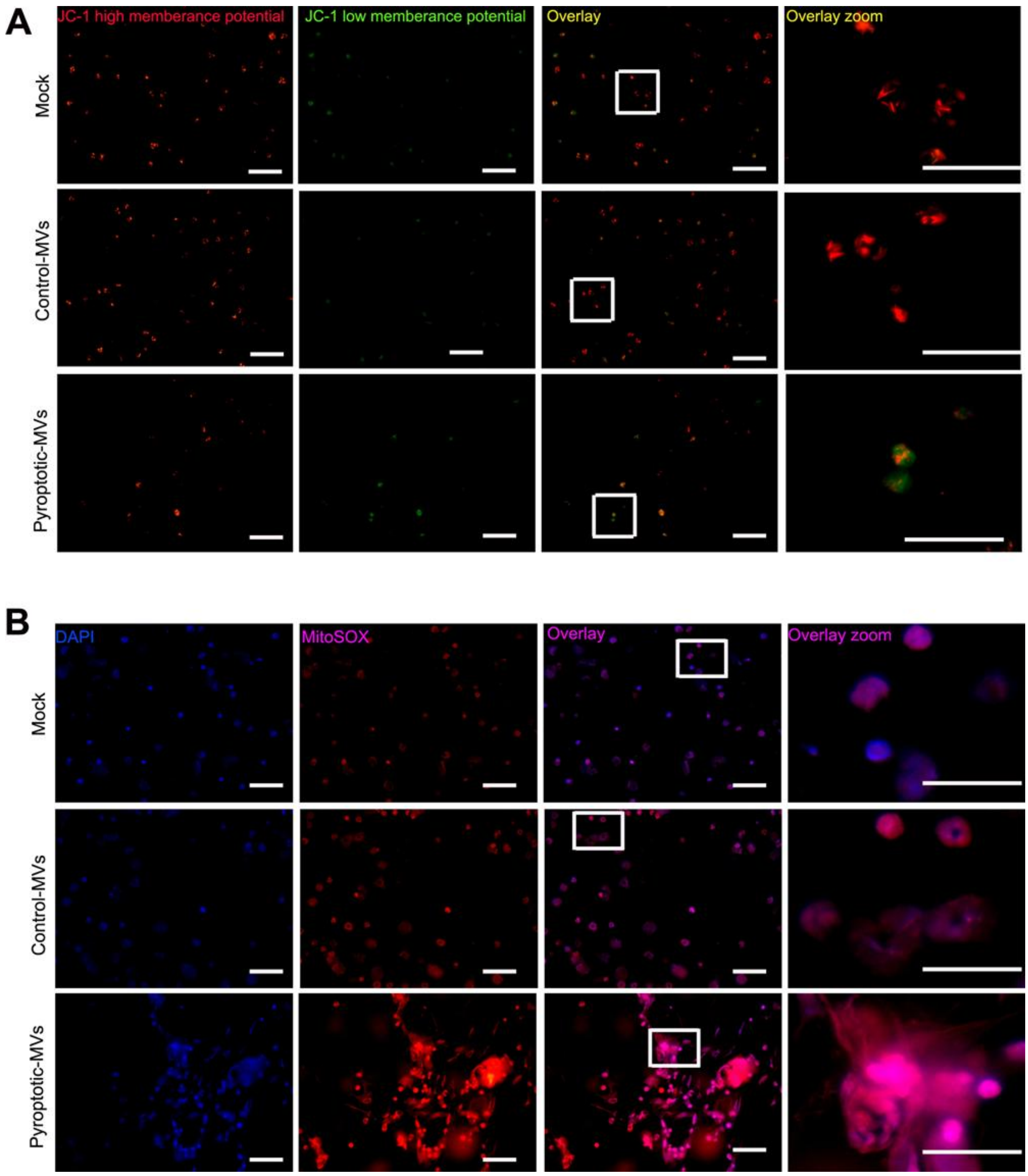

Fig. S4. Pyroptosis Macrophage-derived MVs Altered Mitochondrial Homeostasis in mouse Neutrophils. Mouse neutrophils after exposure to PBS, control macrophage-derived MVs and pyroptotic macrophage-derived MVs for 4 h. (A) ΔΨ of mouse neutrophils was assessed by JC-1staining. JC-1 monomers (green) and aggregates (red) were detected by fluorescence imicroscope. Scale bar, 20 μm. (B) Representative Sytox green fluorescence image for NETs formation. Scale bar, 20 μm.

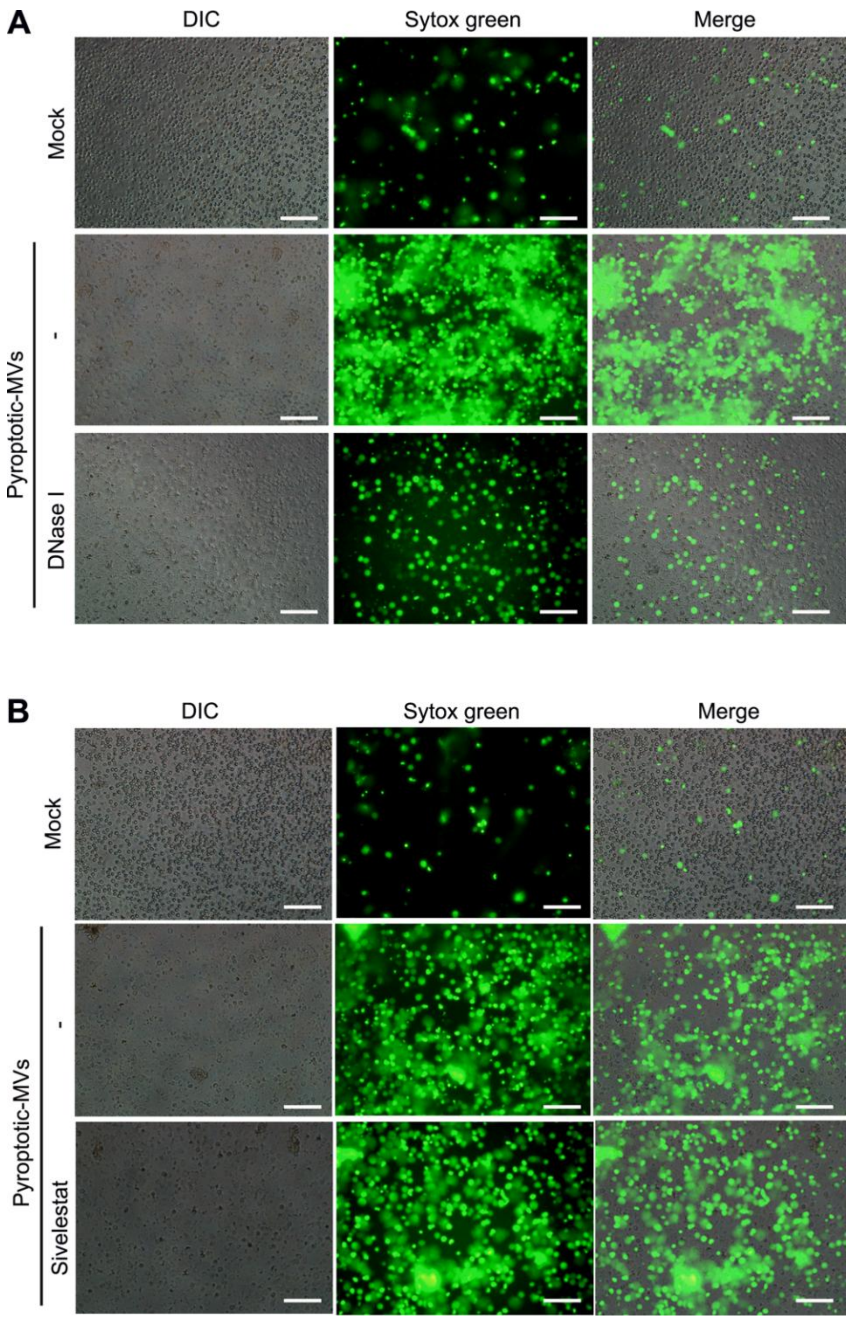

Fig. S5. NETs formation when human neutrophils were exposed to pyroptotic macrophage-derived MVS in the presence of DNaseI and sivelestat. (A) Human neutrophils were treated with pyroptotic macrophage-derived MVs in presence of DNase I (10U/ml) or not for 3-4h. (B) Human neutrophils were treated with pyroptotic macrophage-derived MVs in presence of neutrophil elastase inhibitor Sivelestat (10μM) or not for 3-4h. Scale bar, 50 μm.

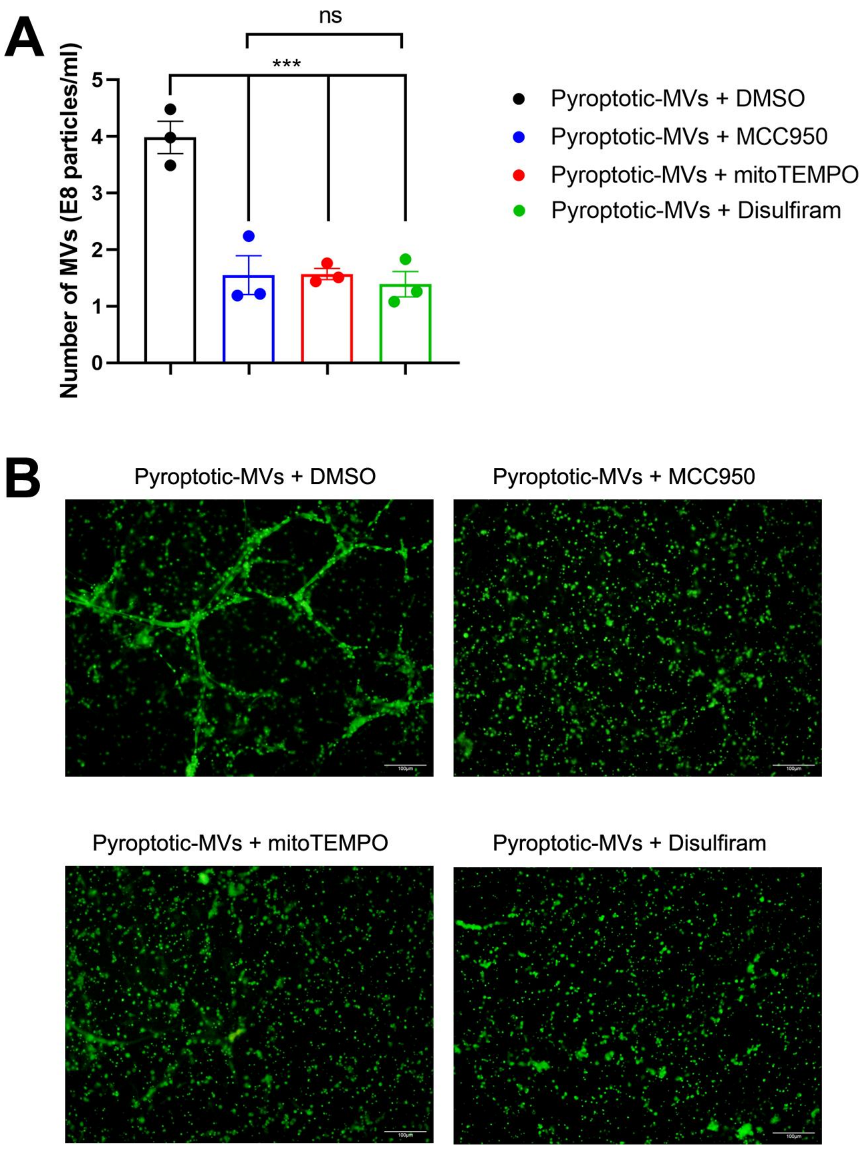

Fig. S6. Inhibition of Pyroptosis and mtROS/GSDMD axis reduced Macrophage-derived MVs and NETs Formation. (A) BMDM were pretreated with DMSO, MCC950 (5 μM), mitoTEMPO (10 μM) or Disulfiram (120 μM) for 1h. To induce BMDM pyroptosis, these pretreated cells were exposed to LPS (500 ng/mL) for 4h, and then nigericin (10 uM) for 1.5h. The culture supernatant was collected for isolating microvesicles. (A) The number of MVs derived from pyroptotic macrophages was measured by NTA (n=3 wells per group). (B) Representative Sytox Green fluorescence image for NETs formation of mouse bone neutrophils (n=3 wells per group).
